# Association between obstructive sleep apnea symptom subtypes and mental health

**DOI:** 10.36416/1806-3756/e20250058

**Published:** 2026-03-05

**Authors:** Daniel Solomons, Mario Henríquez-Beltrán, Montserrat Sanchez, Jorge Jorquera, Gonzalo Labarca

**Affiliations:** 1. Departamento de Enfermedades Respiratorias, Escuela de Medicina, Pontificia Universidad Católica de Chile, Santiago, Chile.; 2. Núcleo de Investigación en Ciencias de la Salud, Universidad Adventista de Chile, Chillán, Chile.; 3. Departamento de Medicina Respiratoria, Clinica Las Condes, Santiago, Chile.; 4. Respiratory, Allergy and Sleep Medicine, Mayo Clinic, Jacksonville, USA.

**Keywords:** Sleep apnea, obstructive, Phenotype, Precision medicine, Mental health

## Abstract

**Objective:**

Obstructive sleep apnea (OSA) of varying severity significantly impacts mental health and quality of life. Recent studies have identified different OSA phenotypes, emphasizing symptom-based patient profiles. We sought to determine the association between symptom-based OSA phenotypes and depression.

**Methods:**

This was an observational study including 1,083 patients (883 men and 200 women) who were part of a previous cohort study and who had been diagnosed with OSA on the basis of an apnea-hypopnea index ≥ 5 events/h on a home sleep apnea test. Sleep-related symptoms were assessed by the Epworth Sleepiness Scale (ESS) and self-reported sleep patterns. Objective respiratory parameters derived through polysomnography were used in order to characterize OSA severity. Depressive symptoms were evaluated with the Beck Depression Inventory (BDI). Cluster analysis defined three groups: nonsleepy patients; patients with disturbed sleep; and patients with excessive daytime sleepiness. Linear regression models were adjusted for age, sex, BMI, respiratory disturbance index, and total sleep time. A sensitivity analysis correlated ESS scores and total sleep time with BDI scores by means of Spearman correlation.

**Results:**

The median respiratory disturbance index was 21.6 events/h (IQR, 12.4-38.1), and the median ESS score was 8.0 (IQR, 5.0-12). A total of 431 patients (39.8%) were nonsleepy, 485 (44.7%) had disturbed sleep, and 167 (14.4%) presented with excessive daytime sleepiness, with a median BDI score of 12 (IQR, 6.0-53.1). Linear regression revealed higher depressive symptoms in patients with disturbed sleep and in those with excessive daytime sleepiness.

**Conclusions:**

Patients with excessive daytime sleepiness and OSA have a higher risk of poor mental health. This underscores the need to address physiological and psychological aspects of OSA for improved patient well-being.

## INTRODUCTION

Obstructive sleep apnea (OSA) is the most common form of sleep apnea^1^ and includes a range of acute symptoms such as disrupted sleep, loud snoring, and gasping for air during sleep. In addition to these direct physical symptoms, there are more long-term symptoms such as fatigue, difficulty concentrating, mood swings, and headaches. Long-term OSA effects pose physiological and psychological risks to patients, including an increased risk of developing depression, although the mechanisms underlying the link between depression and OSA remain unclear.^2^ Depression and OSA have been reported as being highly common in individuals > 65 years of age, with OSA affecting 50% and depression affecting 26%; in addition, 20% of those who have one also have the other.^3^ This overlap between depression and OSA-which also includes anxiety-has been linked to decreased cognitive functioning, including memory and attention.^4^

The diagnosis of OSA is based on the apnea-hypopnea index (AHI),^5^ which measures the number of apneas and hypopneas caused primarily by upper airway obstructions during a night of sleep. Although the AHI is widely used to classify OSA severity, patients with similar AHI values can present with very different symptom patterns and clinical consequences. This heterogeneity has led researchers to look into further methods whereby OSA can be identified and treated, including identification of phenotypic subtypes of OSA through cluster analysis.^6^ ^,^ ^7^ OSA phenotypes, or symptom-based categories of OSA, can be established on the basis of behavioral, demographic, and clinical characteristics such as age, BMI, level of physical activity, and depression, as well as of polysomnographic features and cardiovascular outcomes.^8^ ^,^ ^9^ One study suggested that females with mood disorders be considered a subgroup of OSA patients.^9^ Cluster analyses of this type provide a nuanced insight into OSA symptoms that a standard AHI cannot provide, enhancing the potential for treatment outcomes in a personalized manner.^10^ This can be seen in studies examining the impact of phenotype on OSA treatment with CPAP.^11^ ^,^ ^12^

Establishing a link between OSA and depression provides treatment opportunities because instead of treating OSA with CPAP, adherence to which is not always achieved,^13^ ^,^ ^14^ we can provide treatment for depression as a proxy for OSA treatment, thus improving the quality of life of patients.^15^ However, establishing a clear causal link between OSA and depression has proved difficult,^16^ ^,^ ^17^ with reports of issues regarding the validity and generalizability of findings^18^ and self-report questionnaires overestimating the prevalence of depression in comparison with psychiatric examination, which is the gold standard.^17^

Given that adherence to CPAP treatment is lower in those with depressive symptoms^19^ and that OSA-induced excessive daytime sleepiness is linked to depression,^20^ identifying depressive symptoms in OSA patients is vital to improve diagnosis and treatment, as well as quality of life and overall well-being. 

Although OSA phenotypes have been increasingly recognized, it remains unclear whether symptom-based profiles differ in their association with depression, given that most studies have relied solely on AHI-based severity. We hypothesized that patients with excessive daytime sleepiness and disturbed sleep phenotypes would have higher depressive symptom scores than would nonsleepy patients, even after adjusting for demographic and polysomnographic variables. Therefore, the objective of the present study was to examine the association between symptom-based OSA phenotypes and depressive symptoms. 

## METHODS

**Figure 1:**
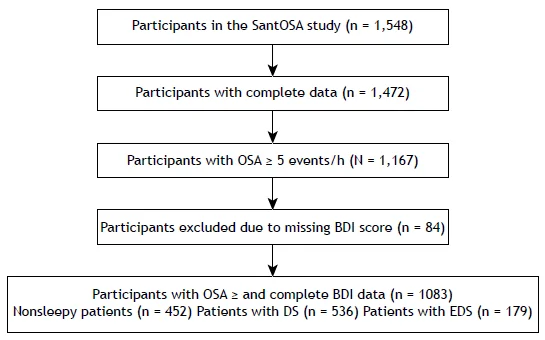
Flow chart of the study pipeline. OSA: obstructive sleep apnea; BDI: Beck Depression Inventorry; DS: disturbed sleep; EDS: excessive daytime sleepiness; and SantOSA: Santiago Obstructive Sleep Apnea.^22^

Data were collected from the Santiago Obstructive Sleep Apnea (SantOSA) clinical database in accordance with the Strengthening the Reporting of Observational Studies in Epidemiology guidelines.^21^ The SantOSA study^22^ was registered in 2019 (ISRCTN62293645), with ethical approval from the Institutional Review Board of *Clinica Las Condes*, located in the city of Santiago, Chile. The SantOSA study^22^ included 1,083 participants > 18 year of age, all of whom underwent a home sleep apnea test (HSAT) in the period between 2009 and 2020 after giving written informed consent. All participants underwent clinical evaluation of sociodemographic characteristics, lifestyle factors (including tobacco and alcohol consumption), comorbidities (including hypertension, diabetes mellitus, coronary heart disease, and COPD), and medication use. For anthropometric measurements, weight and height were recorded after an overnight fast, with the patients in their underwear. The BMI was calculated (in kg/m^2^), and neck circumference was measured at the level of the cricoid with a flexible plastic tape measure. Figure 1 shows the study pipeline. 

At baseline, participants completed a self-report questionnaire assessing their sleep patterns, as well as daytime sleepiness, snoring intensity, witnessed apneas, insomnia, episodes of nocturnal suffocation, and morning headaches. Additionally, we gathered data using the Spanish version of the Epworth Sleepiness Scale (ESS).^23^

All participants underwent an HSAT using a validated type 3 sleep monitoring system (Embletta Multi-Parameter Recorder; Natus Medical, Inc., Middleton, WI, USA). The system recorded nasal airflow (via nasal pressure); thoracic and abdominal respiratory effort (inductance plethysmography); body position; snoring (through an integrated microphone); and pulse oximetry. The device has demonstrated 92.4% sensitivity and 85.7% specificity for detecting OSA when compared with polysomnography in patients with suspected OSA.^24^

The HSAT was performed once at the beginning of the study, and for participants who underwent repeated recordings, only the initial diagnostic test was included in the analysis. All HSAT data were manually scored in accordance with current international guidelines by a respiratory specialist blinded to participant clinical data.^25^ ^,^ ^26^ The following respiratory parameters were extracted: the respiratory disturbance index (RDI), defined as the number of apneas and hypopneas associated with ≥ 3% oxygen desaturation per hour; mean oxygen saturation (SpO_2_); minimum SpO_2_; percent sleep time with SpO_2_ below 90%; and oxygen desaturation index (≥ 3%). OSA severity was classified as mild (an RDI of 5-15 events/h), moderate (an RDI of 15-30 events/h), or severe (an RDI > 30 events/h). 

Mental health was evaluated with the Beck Depression Inventory (BDI),^27^ which is a standardized tool designed to measure the severity of depressive symptoms. The BDI provides insight into the emotional well-being of participants and the presence of depressive symptoms. 

Data analysis included a latent class cluster analysis, which is a statistical method used in order to identify groups of patients with similar symptoms. Patients were divided into three distinct groups: nonsleepy patients; patients with disturbed sleep; and patients with excessive daytime sleepiness. Although the analysis followed the methodology described by Mazzotti et al.,^28^ we excluded the “minimally symptomatic” cluster used in their study. We considered that cluster to be too similar to the “moderately sleepy” group in its outcomes and therefore did not include it in our analysis. The original R code is publicly available at https://github.com/mazzottidr/OSA_Symptom_Subtypes_LCA_Pipeline.git. Fifteen sleep-related questions from the SHHS during the first visit were used for our cluster analysis, with 14 questions being categorized as yes/no questions.^29^ The total ESS score was grouped into ranges: > 10 (nonsleepy patients); 10-15 (patients with disturbed sleep); and > 15 (patients with excessive daytime sleepiness). Additionally, four comorbidities-hypertension, diabetes mellitus, COPD, and coronary heart disease-were included, as reported elsewhere.^30^ The cluster analysis was applied to all OSA patients, as previously defined. The number of clusters was determined on the basis of parsimony criteria, the solution with the lowest Bayesian information criterion being selected for optimal interpretation and analysis, a statistical measure that is used in order to select the best model and that is consistent with the methods used in earlier cluster analyses.^30^

Baseline characteristics were summarized as medians and interquartile ranges for continuous variables and as proportions for categorical variables. The association between symptom subtypes and BDI scores was examined by using multivariate linear regression models: an overall adjusted model; a model including males only; and a model including females only. All models included age, sex, BMI, log-transformed RDI, total sleep time (TST), smoking status (former smoker, current smoker, and unknown/other), and percent sleep time with SpO_2_ below 90% as covariates. 

Regression results are reported as beta coefficients (β) with 95% confidence intervals and values of p. 

A sensitivity analysis was performed to test for interactions between symptom subtypes and age, sex, RDI, BMI, and OSA severity. Additionally, Spearman correlation analysis was performed to evaluate the relationship between BDI scores, ESS scores, and TST. 

A value of p < 0.05 was considered significant. All analyses were performed with R software, version 4.5.2 (R Foundation for Statistical Computing, Vienna, Austria). 

## RESULTS

**Figure 2:**
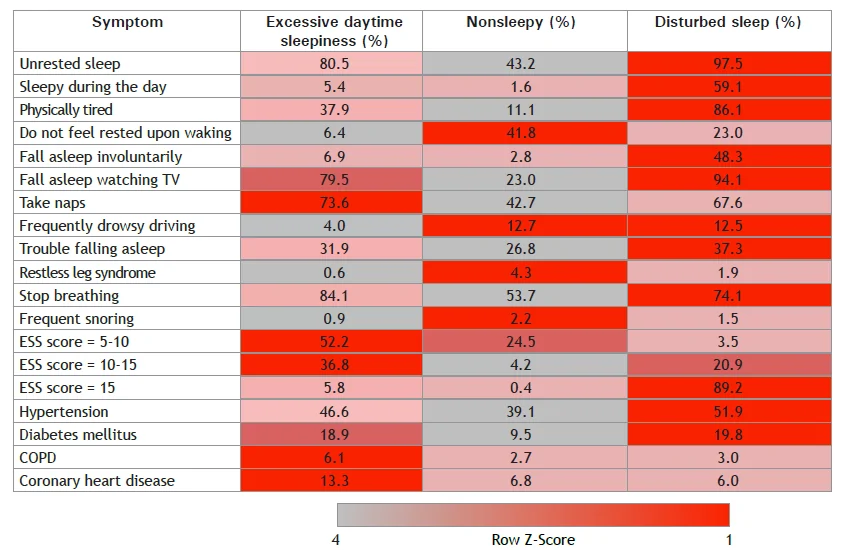
Latent class analysis results showing patient answers to the questions and the corresponding heat map. EDS: excessive daytime sleepness.

**Figure 3:**
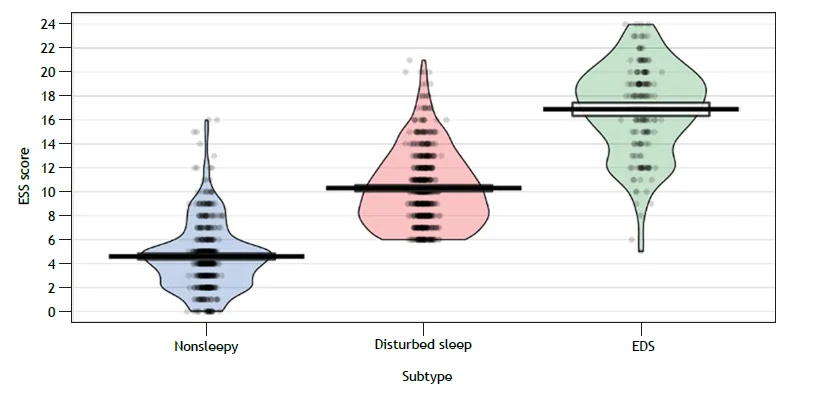
Epworth Sleepiness Scale (ESS) scores across different symptom subtypes: nonsleepy patients; patients with disturbed sleep; and patients with excessive daytime sleepiness (EDS).

A total of 1,083 participants were included in the analysis. On the basis of our latent class analysis, three symptom-based subtypes were identified: nonsleepy patients (39.8%); patients with disturbed sleep (44.7%), and patients with excessive daytime sleepiness (14.4%; Table 1). The median RDI was 21.6 events/h (IQR, 12.4-38.1), the median ESS score was 8.0 (IQR, 5.0-12), and the median BDI score was 12 (IQR, 6.0-43.1). Patient answers to the questions and the corresponding heat map are shown in Figure 2. ESS scores were significantly higher in the patients with disturbed sleep and in those with excessive daytime sleepiness (Figure 3). 

**Table 1:** Baseline characteristics of the study population.^a^

	Nonsleepy patients (n = 431)	Patients with disturbed sleep (n = 485)	Patients with excessive daytime sleepiness (n = 167)
Age, years	54 [42-64]	54 [43-63]	52 [44-60]
Male	354 (82.1%)	401 (82.7%)	128 (76.6%)
BMI, kg/m^2^	29.2 [26.7-31.8]	30.4 [27.5-33.7]	31.7 [28.3-34.7]
Current smoker	116 (29.9%)	102 (21%)	26 (15.6%)
ESS score	4.0 [3.0-6.0]	10.0 [8.0-12.0]	17.0 [14.0-19.0]
BDI score	8.0 [4.0-18.0]	15.0 [7.0-43.2]	27.0 [10.0-55.0]
RDI, events/h	17.4 [10.8-28.4]	25.1 [13.3-42.2]	32.4 [16.2-54.6]
T90	3.5 [0.7-12.7]	5.9 [1.2-21.4]	9.0 [1.5-29.8]
TST, h	6.5 [6.0-7.5]	6.5 [6.0-7.5]	6.0 [5.0-7.0]

ESS: Epworth Sleepiness Scale; BDI: Beck Depression Inventory; RDI: respiratory disturbance index; T90: percent sleep time with SpO_2_ below 90%; and TST: total sleep time. ^a^Data expressed as n (%) or median [IQR].

In comparison with the group of nonsleepy patients, the disturbed sleep and excessive daytime sleepiness subtypes were independently associated with significantly higher BDI scores after adjustment for covariates. In the fully adjusted model, BDI scores were on average 13.2 points higher (95% CI, 10.0-16.4; p < 0.001) in the disturbed sleep group and 25.1 points higher (95% CI, 20.6-29.6; p < 0.001) in the excessive daytime sleepiness group (Table 2). No significant interactions were observed between symptom subtypes and age, sex, or OSA severity. 

**Table 2:** Multivariate linear regression models evaluating the association between symptom-based obstructive sleep apnea phenotypes and depressive symptoms (with the Beck Depression Inventory score as the dependent variable) in the sample as a whole and stratified by sex.

Regression model	Male/Female			Male			Female		
Predictor	Estimate*	CI	p**	Estimate*	CI	p**	Estimate*	CI	p**
(Intercept)	−45.09	−58.28 to −31.89	< 0.001	−40.65	−55.55 to −25.74	< 0.001	−52.36	−82.54 to −22.18	0.001
LCA 5 cat [DS]^†^	13.21	10.0-16.4	< 0.001	11.27	7.76-14.77	< 0.001	21.24	13.48-29.00	< 0.001
LCA 5 cat [EDS]^†^	25.09	20.6-29.57	< 0.001	24.97	19.93-30.01	< 0.001	26.30	16.42-36.18	< 0.001
Age	0.18	0.06-0.29	0.003	0.17	0.04-0.30	0.008	0.17	−0.12 to 0.46	0.256
Sex^††^	5.86	1.92-9.80	0.004						
BMI	0.14	−0.18 to 1.45	0.381	1.08	0.71-1.45	< 0.001	1.38	0.77-1.99	< 0.001
Current smoker	8.32	4.97-11.68	< 0.001	9.20	5.49-12.91	< 0.001	4.77	−3.16 to 12.69	0.237
Former smoker	−17.87	−21.61 to −14.12	< 0.001	−17.13	−21.19 to −13.07	< 0.001	−20.34	−30.10 to −10.55	< 0.001
Unknown/other	−6.30	−9.64 to −2.97	< 0.001	−5.71	−47.18 to 46.28	0.983	−2.75	−51.78 to 46.28	0.912
RDI [log]	0.16	−0.16 to 0.48	0.329	0.12	−0.58 to 0.46	0.128	0.19	−0.81 to 0.58	0.941
T90	−0.05	−0.10 to −0.00	0.048	−0.01	−0.08 to 0.08	0.848	−0.16	−0.29 to −0.02	0.029
TST	2.14	1.16-3.12	< 0.001	1.63	0.49-2.76	0.005	3.56	1.68-5.64	< 0.001
N	1,083			883			200		
R^2^/adjusted R^2^	0.321/0.314			0.301/0.293			0.373/0.340		

TST: total sleep time; T90: percent sleep time with SpO_2_ < 90%; RDI: respiratory disturbance index; LCA: latent class analysis; DS: disturbed sleep; and EDS: excessive daytime sleepiness. *Unstandardized beta coefficient. **Values of p < 0.05 were considered significant. ^†^Reference: nonsleepy patients. ^††^Reference: male.

The Spearman correlation between BDI and ESS scores was ρ = 0.40 (p < 0.001), indicating a moderate positive association, whereas the correlation between BDI and TST was ρ = 0.14 (p = 0.03), suggesting a weak relationship. 

In sex-stratified analyses, associations were consistent for males and females. Among males, adjusted β coefficients were 11.3 (95% CI, 7.7-14.7; p < 0.001) for the disturbed sleep subtype and 24.9 (95% CI, 19.9-30.1; p < 0.001) for the excessive daytime sleepiness subtype. Among females, corresponding β coefficients were 21.2 (95% CI, 12.4-29.0; p < 0.001) and 26.3 (95% CI, 16.4-36.2; p < 0.001), respectively. 

## DISCUSSION

The present study highlights the association between excessive daytime sleepiness and OSA with an elevated risk of depressive symptoms, as indicated by linear regression analyses. The link was further clarified by clustering patient characteristics to identify distinct phenotypic subgroups; in other words, separate symptom-based OSA categories. 

Given the well-documented high prevalence of depressive symptoms in OSA patients,^2^ our study sought to provide a deeper understanding of this relationship; specifically, whether OSA of varying severity significantly impacts mental health and quality of life. Our findings are consistent with those of previous studies reporting a connection between OSA, daytime sleepiness, and depressive symptoms.^8^ ^,^ ^9^ However, the novelty of our study lies in its targeted focus on depression as the primary outcome rather than on broader symptom-based classifications. By isolating depressive symptoms as a key variable, we provide new insights into how distinct OSA phenotypes-particularly those characterized by excessive daytime sleepiness or disturbed sleep-may be differentially associated with mood disturbances. This phenotypic framework supports the growing view that OSA is not a uniform disorder but a heterogeneous condition with diverse clinical expressions, each potentially linked to different neurobehavioral and physiological consequences. 

In our sensitivity analyses, we found no interaction between depressive symptoms and age, sex, or OSA severity. This indicates that the relationship between OSA and depression may occur independently of demographic factors or disease severity, suggesting that even individuals with mild or moderate OSA can experience notable depressive symptoms. These results highlight the importance of incorporating mental health assessment into the routine evaluation of OSA patients, regardless of severity. The weak correlation observed between depressive symptoms and TST suggests that sleep duration alone is unlikely to explain mood alterations in patients with OSA. Instead, the impact of sleep fragmentation, intermittent hypoxia, and their downstream neurobiological effects appears to play a more substantial role in influencing mood and energy regulation. From a mechanistic standpoint, recurrent nocturnal hypoxia and sleep fragmentation can impair monoaminergic neurotransmission, alter hypothalamic-pituitary-adrenal axis regulation, and promote systemic inflammation, all of which are implicated in the pathophysiology of depression.^4^ ^,^ ^9^ These pathways may explain why certain OSA phenotypes, particularly those marked by daytime sleepiness and low energy, display higher depressive symptom scores. 

Overall, recognizing and addressing depressive symptoms in OSA patients is crucial for improving overall health outcomes. Identifying the subgroup of OSA patients most vulnerable to mood disturbances may support the development of more tailored interventions and early prevention strategies. Increased clinical awareness of this phenotype-oriented approach will help optimize treatment and move toward more personalized care for patients with OSA.^31^ ^,^ ^32^

Despite the promising results, some factors should be considered. First, the sleep behavior data were based on self-report questionnaires, which may introduce response bias or inaccuracies in participant reporting connected to the subjective nature of the symptoms reported. Second, although depressive symptoms were assessed with the BDI, the fact that a formal clinical diagnosis of depression was not made weakens the robustness of the mental health findings. Third, because of the cross-sectional design of our study, in which we examined data from one point in time, we cannot establish a definitive causal relationship between clinical depression and OSA on the basis of our findings alone, despite the fact that we showed how OSA phenotypes such as daytime sleepiness may contribute to depressive symptoms. Finally, because 82% of our cohort was male, the present study has limited applicability to the female population. 

From a clinical perspective, our findings highlight the need for systematic screening for depressive symptoms in patients with disturbed sleep and excessive daytime sleepiness phenotypes, regardless of AHI severity. Phenotype-based assessment might be better to identify patients at psychological risk than traditional metrics alone. This approach supports a more personalized management strategy in OSA, integrating mental health evaluation into routine care. Moreover, the stronger association observed in women suggests that sex-specific screening protocols are warranted. Incorporating phenotype-driven mental health assessment into diagnostic and therapeutic pathways could improve adherence to CPAP and overall patient outcomes. 

In conclusion, our study underscores the importance of recognizing depressive symptoms within specific phenotypic, symptom-based subgroups of OSA patients, highlighting a critical overlap between these two conditions. Our findings suggest that considering depression as a key factor in OSA diagnosis and treatment could improve clinical outcomes. By identifying at-risk subgroups, we contribute to the broader effort to refine diagnostic criteria for OSA and emphasize the need for comprehensive, individualized treatment strategies. 
